# Effects of individualized dietary programs provided by nurses on nutrition and micro-inflammation of patients undergoing peritoneal dialysis (PD): A non-randomized controlled study

**DOI:** 10.1097/MD.0000000000040724

**Published:** 2024-11-29

**Authors:** Peng Shu, Yani Lv, Li Guo, Fang Xu

**Affiliations:** a The Central Hospital of Wuhan, Tongji Medical College, Huazhong University of Science and Technology, Wuhan, Hubei Province, China.

**Keywords:** dietary, micro-inflammatory, nursing, nutrition status, peritoneal dialysis

## Abstract

This study aimed to assess the effects of individualized dietary programs provided by nurses on the nutrition and micro-inflammation of patients undergoing peritoneal dialysis (PD). This study employed the convenience sampling method for selecting participants. Accordingly, 60 patients undergoing PD who visited a PD center from January to June 2022 were selected as the control group, and 60 patients undergoing PD who visited the same PD center from November 2022 to April 2023 were selected as the intervention group. Participants in the intervention group received individualized dietary nutrition programs, whereas those in the control group received general nursing programs. The nutritional and micro-inflammatory statuses of participants in both groups were assessed after 6 months. The nutritional status was assessed based on nutritional scores and blood tests (red blood cells, pre-albumin, albumin, ultrasensitive C-reactive protein, white blood cells, lymphocytes, neutrophils, and globulins), and the micro-inflammatory status was assessed based on blood tests. After 6 months, the intervention group outperformed the control group in terms of lymphocyte percentage, ultrasensitive C-reactive protein level, lymphocyte count, and white blood cell count (*P* < .05). In addition, globulin, preprotein, albumin, and hemoglobin levels, and red blood cell count were higher in the intervention group compared to the control group (*P* < .05). The results also showed a lower prevalence of peritonitis in the intervention group (*P* < .05). The participants in the intervention group obtained lower nutritional scores than those in the control group (*P* < .05). There were no significant differences between the pre- and post-intervention indexes in the control group (*P* > .05). In contrast, the studied indexes significantly improved in the intervention group (*P* < .05). The study findings suggested that individualized dietary programs provided by nurses can improve malnutrition, micro-inflammatory, and peritonitis in patients with PD.

## 1. Introduction

End-stage kidney disease (ESKD) has become a disease that seriously affects patients’ health and quality of life.^[1]^ Kidney transplantation, hemodialysis, and Peritoneal dialysis (PD) are the current treatment options for ESKD. However, PD is the preferred renal replacement therapy because of its simplicity, affordability, hemodynamic stability, and ability to preserve residual renal function.^[2]^ The main reasons for malnutrition among patients undergoing PD are: (1) decreased appetite following the entry of a high amount of PD fluid into the abdominal cavity, which induces fullness and reduces food intake; (2) various metabolites in the body of patients with ESRD stimulate the gastrointestinal tract, resulting in gastrointestinal dysfunction and reducing the patient’s appetite; (3) acidosis causes various organisms to produce various inflammatory factors, accelerating the decomposition of proteins; (4) infection can accelerate the consumption of protein in patients’ body, resulting in malnutrition; (5) inadequate dialysis leads to nausea, vomiting, regurgitation, and other symptoms, resulting in reduced food intake and malnutrition; (6) loss of albumin in dialysate.^[3,4]^ Therefore, it is important for nurses to improve how they use dietary programs to increase the appetite and food intake of patients undergoing PD.

Many studies have shown that ESKD is a systemic chronic inflammatory state and several factors lead to a micro-inflammatory state in patients with ESKD. Malnutrition and micro-inflammation seriously affect the prognosis and quality of life of patients undergoing PD.^[5–7]^ Comprehensive management is essential for addressing micro-inflammatory status and malnutrition in patients undergoing peritoneal dialysis. Effective management strategies encompass optimizing dietary nutrient intake, appropriately treating metabolic disorders (such as metabolic acidosis and hormone deficiencies), controlling inflammation, and refining dialysis regimens. Additionally, it is important to preserve residual renal function and encourage appropriate exercise.^[8–12]^ Some studies have shown that pharmacological treatments combined with a specific diet can achieve positive therapeutic outcomes.^[13]^ However, a few studies have assessed how dietary interventions can improve nutritional status and micro-inflammation in patients undergoing PD. Therefore, this study aimed to assess the effects of individualized dietary programs provided by nurses on the nutritional status and micro-inflammation of patients undergoing PD.

### 1.1. Information and methods

#### 1.1.1. Study design: this study was a non-randomized controlled study

This non-randomized controlled trial employed convenience sampling for selecting participants. Accordingly, 60 patients undergoing PD who visited the PD center of the Central Hospital of Wuhan from January to June 2022 were recruited as the control group, and 60 patients undergoing PD who visited the same center from November 2022 to April 2023 were selected as the intervention group. Patients were treated with 1.5 or 2.5 mmol/L of glucose-based dialysis fluid for continuous ambulatory peritoneal dialysis. The participants attended 4 dialysis sessions per day, with a 4-hour interval between each session, for 6 months. The dialysate volume was 8 L per day.

### 1.2. Methods

#### 1.2.1. Establishment of a research team

The research team was led by an author and included a PD specialist, a PD nurse, a research nurse, and 2 chronic kidney disease management nurses. The PD specialist and nurse were responsible for assessing the patient’s condition and adjusting the treatment plan. The chronic kidney disease management nurse was responsible for the implementation of dietary and nutritional measures and follow-up management. The research nurse was responsible for the quality control of the administration of nursing measures.

#### 1.2.2. Inclusion criteria were as follows

(1) Patients visited the studied hospital; (2) patients with a conscious state; (3) patients undergoing continuous ambulatory peritoneal dialysis-based dialysis, (4) undergoing dialysis 4 times a day with a 4-hour abdominal retention time; (5) patients who used the PD solution (lactate-G1.5% and G2.5%); (6) patients who were willing to participate in the study and signed the informed consent form; (7) patients with an expected survival time of more than 6 months; (8) patients aged 18 to 75 years; (9) a dialysis vintage of 0 to 240 months; and (10) patients’ peritoneal equilibration test: *K*_*t*_/*V* ≥ 1.7.

#### 1.2.3. Exclusion criteria were as follows

(1) Patients who were also on hemodialysis; (2) patients who were also suffering from tumors and other physical conditions; (3) patients who could not complete the study; and (4) patients who needed enteral feeding.

This study was approved by the Ethics Committee of our Hospital (WHZXKYL2022-019). In addition, this study was also registered with the Chinese Clinical Trial Registry (ChiCTR2300078936).

The sample size was calculated using the following formula: n_1_ = n_2_ = 2(t_α_+t_β_)^2^S^2^/δ^2^.

In this formula, S denotes the overall standard deviation of the 2 groups and δ represents the difference between the overall means of the 2 groups, taken as α = 0.05 and β = 0.10. Based on the *preliminary preexperiment and review of relevant literature.*

S = 0.15, δ = 0.1, t_α_ = 1.960, and t_β_ = 1.282, the sample size was calculated as 48 in each group. Considering an attrition rate of 10% until the follow-up stage and other factors, the final sample size was determined to be 60 in each group.

#### 1.2.4. Control group

Sixty patients undergoing PD who visited the studied PD center from January 2022 to June 2022 were recruited as the control group. The research team developed a health management file for each participant in this group. The participants were instructed on the types of foods they could and could not eat based on the results of blood tests and nutritional assessments. They were also instructed to follow a diet rich in high-quality proteins, such as milk, eggs, and lean meat. The participants were asked to avoid high-phosphorus food items such as pickles and burgers. They were also instructed in some cooking techniques, such as boiling the vegetables before consumption, to reduce the potassium content of their diets.

#### 1.2.5. Intervention group

**Figure 1.**
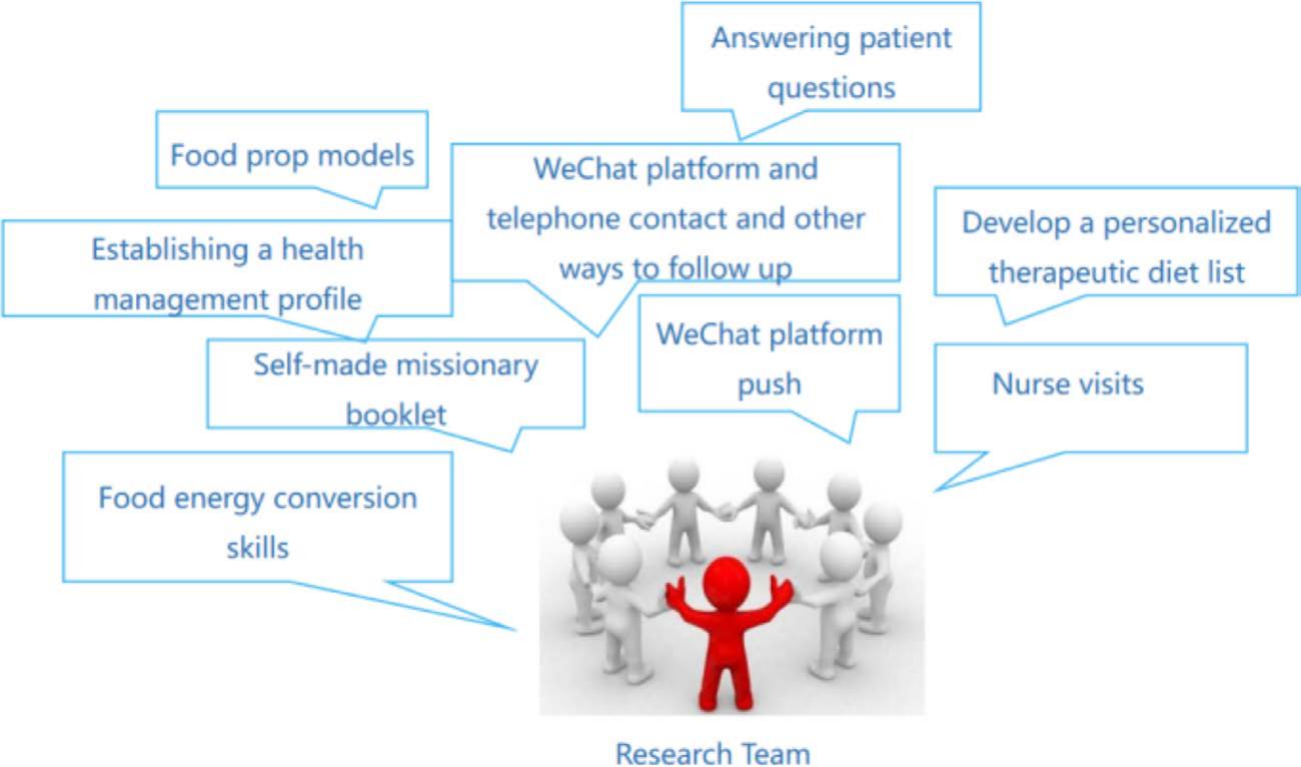
Study Team intervention components.

Sixty patients undergoing PD who visited the studied PD center from November 2022 to April 2023 were recruited as the intervention group. After entering the study, the research team developed a health management file for each participant in this group. The medical and nursing staff of the research team followed the patients via WeChat and phone calls as part of routine care measures determined by the department. The research team provided appropriate guidance to the patients in this group and their support persons. A dedicated person was responsible for monitoring each participant’s diet, physical activity, and health status based on the following protocol (Fig. 1):

(1) The PD nurse was responsible for developing a diet list and determining how much protein and calories are needed for each participant. The international standard recommended calculation method for Chinese individuals is known as “standard body mass”^[14,15]^:

Standard body mass for males = [height (cm) ‐ 100] *×* 0.9 (kg);

Standard body mass for females = [height (cm) ‐ 100] *×* 0.9 (kg) − 2.5 (kg);

Protein intake: 0.8 to 1.0 g/kg;

Total calories: 125.5 to 140 kJ/kg.

The PD nurse and the dietitian found some food calories from books that could help them serve something to stimulate the appetite of participants. They could then collaborate to develop an individualized diet plan for each participant.

(2) The research team discussed and developed appropriate measures to address the problems related to the complications of participants. The main content was as follows:
  1- Knowledge about the disease: The researcher organized a group of participants to brief them on the causes of peritonitis, malnutrition, and micro-inflammation among patients undergoing PD, their negative effects on body organs, and strategies to effectively control the related complications.
  2- The participants were provided with the appropriate guidelines on protein-rich diets: The research team utilized the food prop model to impart diet-related knowledge about malnutrition and other complications to patients and their support persons. The research team provided diet-related knowledge to the participants and their support persons to increase their awareness of the importance of diet therapy. The research team provided the participants with the corresponding guidance on the disease, diet, and lifestyle via WeChat once a week. The research team formulated a personalized protein-based diet and calculated the daily protein and energy requirements for each participant. Members calculated the daily protein and energy consumption requirements for patients, considering their dietary structure and personal habits. They also formulated a focused diet list to match the personalized treatment of participants. They instructed the participants and their support person to prepare food scales and calorie tables at home and improve simple food and energy conversion skills.
  3- Remote guidance: The research team monitored the participants weekly through telephone or WeChat to ensure their adherence to the diet, correct their misconceptions, answer their questions, and solve their possible problems.
  4- The research team evaluated the nutritional status of the participants in the intervention group at the PD clinic each month. Based on their albumin level and nutritional status score, the research team also calculated their daily protein and calorie intake. The medical and nursing team provided dietary guidance according to the daily dietary habits of participants. For example, a participant was recommended to have 5 g of steamed buns and 50 mL of milk for breakfast, 10 g of rice and 20 g of lean meat for Chinese food, 20 g of noodles, and 15 g of egg white for dinner. This balanced diet provided the participant with enough calories and protein (Supplementary File 1, Supplemental Digital Content, http://links.lww.com/MD/O59).
(3) The participants were asked to visit the hospital each month to check and ensure their adherence to the personalized dietary checklist.

### 1.3. Observation index

Participants in both groups were followed for 6 months. The modified quantitative subjective global assessment (MQSGA) was used to evaluate the nutritional status of the 2 groups. Red blood cells, pre-albumin, albumin, ultrasensitive C-reactive protein (CRP), white blood cells (WBC), lymphocytes, neutrophils, globulins, and the incidence of peritonitis were measured. Peritonitis was confirmed as follows: (1) the patient has abdominal pain and/or turbidity in the peritoneal dialysis effluent; (2) a positive leukocyte count of >100/µL or >100 × 10^6^/L in the peritoneal dialysis effluent (residency time > 2 h); and (3) a positive microbiological culture test for peritoneal dialysis effluent. The obtained data were compared between the 2 groups of patients.

Blood tests were conducted in a laboratory of a tertiary care hospital in Wuhan. Nutritional status was assessed based on nutritional scores and blood tests, and micro-inflammatory status was assessed based on blood tests.

Globulin is a liver function test indicator that is produced by the immune system and found primarily in cells and fluids of the body. This indicator represents the immunity status of patients, as low globulin levels suggest that patient’s immunity has been compromised.^[15]^

The liver produces proproteins, also known as pre-serotonin, which are thyroxine transporter proteins. Preprotein is clinically important because it reflects the patient’s nutritional status, and reduced preprotein levels indicate that the patient is malnourished.^[16]^

MQSGA represents changes in body mass, dietary intake, gastrointestinal symptoms, nutrition-related body functions, comorbidities, subcutaneous fat, and muscle consumption. The total score on this tool ranges between 7 and 35, and higher scores indicate a poorer nutritional status. Scores ≤10, 1l to 20, and 21 to 35 represent a normal nutritional status, mild to moderate malnutrition, and severe malnutrition, respectively.^[17]^

Height, body weight, and anthropometric measurements were conducted by the peritoneal dialysis nurse specialist. Biceps skinfold and triceps skinfold thicknesses were measured using a conventional skinfold caliper, following standard techniques. Mid-arm circumference was measured using a plastic tape. Mid-arm muscle circumference was calculated using the following formula: mid-arm muscle circumference = mid-arm circumference ‐ (3.1415 × triceps skinfold). All anthropometric measurements were conducted on the nonaccess arm. Body mass index was calculated as the ratio of post-hemodialysis body weight in kilograms to the square of height in meters (kg/m²).

### 1.4. Statistical methods

Data were analyzed using SPSS-19. A normality test was conducted. Normally distributed measures are expressed as ($\bar{\chi }\pm s$). Count data were analyzed using the chi-square test, and measurement data were compared using *t* test. The test level was α = 0.05, and *P* < .05 indicated a statistically significant difference.

## 2. Results

### 2.1. General findings

**Figure 2.**
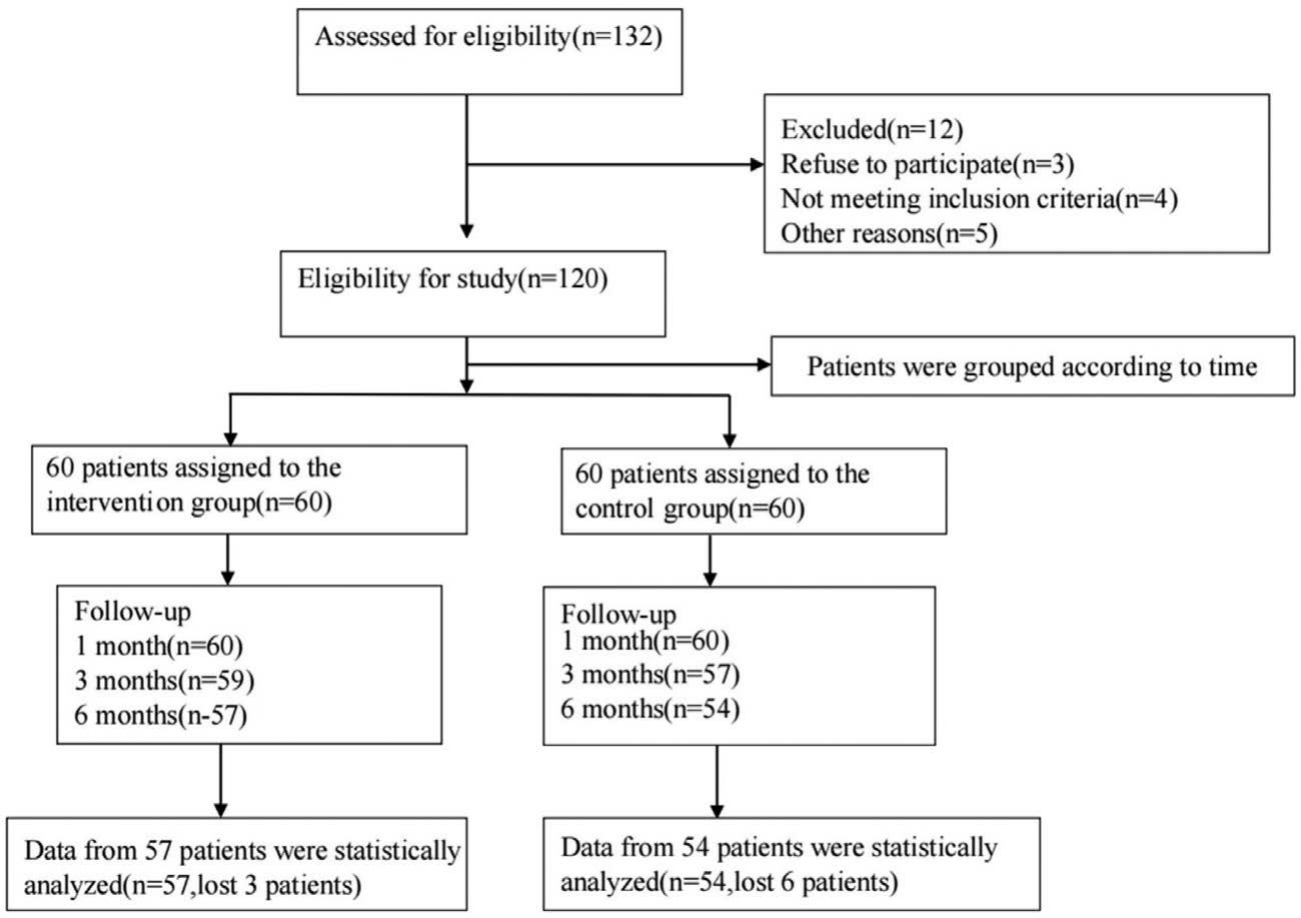
Study design flow chart.

This study was conducted on 120 participants (60 in each group). Six participants from the control group and 3 participants from the intervention group were excluded from the study. The sampling process is outlined in Figure 2. The data showed no significant differences between the 2 groups in terms of demographics (Table 1).

**Table 1:** A comparison between the 2 groups of participants in terms of demographics.

Projects	Intervention group (n = 57)	Control group (n = 54)	*T*/*χ*^2^	*P*-value
Gender			1.477	.22
Male	34	26		
Female	23	28		
Age(years)	59.58 ± 21.47	60.30 ± 13.73	0.288	.77
Underlying diseases			1.100	.34
Diabetic nephropathy	12	13		
Hypertensive nephropathy	32	30		
Chronic glomerulonephritis	13	11		
Education attainment			1.015	.45
Primary school	2	3		
Junior high school and junior college	43	40		
High school and above	12	11		
Duration of dialysis (months)	30.42 ± 12.51	29.65 ± 9.95	0.358	.72

Among the 132 patients who were screened, 120 patients were considered eligible and then they were randomly assigned to the intervention group and control groups. Among them, 57 participants in the intervention group and 54 participants in the control group finished the study.

### 2.2. The 2 groups of participants were compared after 6 months in the micro-inflammatory and nutrition-related indicators (Table 2)

After 6 months, the percentage of lymphocytes, CRP level, lymphocyte count, and WBC improved in the intervention group compared to the control group (*P* < .05). The globulin, preprotein, albumin, hemoglobin, and red blood cells levels were also higher in the intervention group (*P* < .05). The incidence of peritonitis was lower in the intervention group compared to the control group (*P* < .05). Participants in the intervention group exhibited lower nutritional scores than those in the control group (*P* < .05). The post-intervention indexes significantly improved in the intervention group compared to the control group (*P* < .05).

**Table 2:** A comparison between the 2 groups in the pre- and post-intervention indexes.

Projects	Intervention group (n = 57)		Control group (n = 54)		*T*/*χ*^2^	*P*-value
	Pre-intervention	Post-intervention	Pre-intervention	Post-intervention		
Neutrophils (%)	68.73 ± 11.966	69.68 ± 9.819*	69.87 ± 9.616**	76.31 ± 6.294***	4.526	<.001
WBC (10^9^/L)	6.87 ± 2.216	6.66 ± 2.208*	6.67 ± 1.954**	8.93 ± 2.056***	5.589	<.001
Hemoglobin (g/L)	101.16 ± 17.318	99.91 ± 18.212*	99.74 ± 22.610**	90.19 ± 11.902***	3.331	.001
CRP (mg/L)	7.21 ± 14.517	0.38 ± 0.432*	7.21 ± 21.30**	1.83 ± 2.671***	2.756	.006
Albumin count (g/L)	31.56 ± 4.589	34.12 ± 4.415*	37.18 ± 4.423**	30.36 ± 4.331***	4.538	<.001
Lymphocyte (10^9^/L)	1.29 ± 0.592	1.24 ± 0.438*	1.23 ± 0.555**	1.59 ± 0.404***	4.332	<.001
Globulin (g/L)	32.10 ± 8.289	32.10 ± 8.289*	30.31 ± 6.370**	24.85 ± 3.505***	5.938	<.001
Pre-albumin (mg/L)	294.65 ± 92.693	333.37 ± 49.034*	281.67 ± 82.529**	301.78 ± 87.080***	2.371	.02
RBC (10^12^/L)	3.44 ± 0.776	3.68 ± 0.816*	3.31 ± 0.712**	3.33 ± 0.533***	3.235	.002
Nutrition score	9.95 ± 2.642	8.52 ± 1.062*	11.00 ± 3.041**	11.19 ± 2.869***	6.034	<.001
Peritonitis (%)		9 (15.8)		16 (29.6)	4.878	.02

CRP = C-reactive protein, MQSGA = modified quantitative subjective global assessment, WBC = white blood cells.

The *P*-value indicates the post-intervention comparison between the control and intervention groups.

* Indicates that after the intervention, the indicators were compared between the intervention groups *P* < .05.

** Indicates that before the intervention, the comparison of indicators between patients in the control group and the intervention group *P* > .05.

*** Indicates that after the intervention, the comparison of the indicators of the patients in the control group *P* > .05.

### 2.3. Correlation analysis between nutritional scores and inflammation (Table 3)

The results revealed that patients’ hematologic inflammatory factors ((WBC (10^9^/L), CRP (mg/L), lymphocyte (10^9^/L), and neutrophils (%)) positively correlated with nutritional scores (*P* < .05). Furthermore, blood test nutritional indicators (albumin count (g/L), globulin (g/L, hemoglobin (g/L)) negatively correlated with nutritional scores (*P* < .05). No correlation was observed between WBC and nutritional scores (*P* > .05).

**Table 3:** Correlation analysis between nutritional scores and inflammation.

Projects	MQSAG	
	*R*-value	*P*-value
Neutrophils (%)	0.274	.024
WBC (10^9^/L)	0.198	.042
Hemoglobin (g/L)	‐0.959	.003
CRP (mg/L)	0.977	.036
Albumin count (g/L)	‐0.978	.035
Lymphocyte (10^9^/L)	0.965	.004
Globulin (g/L)	‐0.278	.027
Pre-albumin (mg/L)	‐0.756	.023
RBC (10^12^/L)	0.598	.075

CRP = C-reactive protein, MQSGA = modified quantitative subjective global assessment, RBCs = red blood cells, WBC = white blood cells.

## 3. Discussion

### 3.1. Analysis of the effect of dietary and nutritional nursing on the nutritional status of patients undergoing PD

A retrospective study showed that peritonitis, malnutrition, and cardiovascular disease are the main causes of death among patients undergoing PD.^[18]^ According to Kolak et al, patients with PD experience malnutrition and insufficient dietary intake, and there is a correlation between nutritional intake and patients’ 10-year survival.^[19]^ The development of individualized dietary guidelines and structured nutritional support for PD patients by nurses can prevent peritonitis, malnutrition, and mortality.^[20]^ Yixin Luo et al demonstrated that a nurse-led team providing food-switching care measures to patients undergoing PD can effectively improve the nutritional status of patients, which is consistent with the findings of this study.^[21]^

However, existing nutritional and dietary guidance for dialysis patients in China is focused on hemodialysis patients, while patients undergoing PD spend most of their time on home self-care and cannot communicate with healthcare providers in a timely manner. Many patients suffer from nutritional disorders due to inappropriate dietary structure, substandard disinfection of the home environment, and poor hand hygiene, resulting in peritonitis. A nurse-led home follow-up program can effectively improve PD patients’ self-care practices, reduce dialysis-related complications, and improve their quality of life.^[22]^

The results of this study showed that the general out-of-hospital extended care had no significant effect on the nutritional status and micro-inflammatory status of participants. However, nutritional care was effective in improving the nutritional deficiencies and micro-inflammatory status (*P* < .05). This was due to the focus of PD patients on home treatments, which prevented them from effectively completing the care plan developed by nurses. Therefore, nurses should increase patients’ awareness of self-care practices and emphasize the importance of nonpharmacological treatments such as diet and exercise during the interdialysis period to encourage them to actively engage in self-care. Xinjuan Tao et al reported that reasonable physical activity can improve the nutritional status and prognosis of patients.^[23,24]^ A study by Peng et al also showed that nursing management of PD patients can improve the nutritional status of patients.^[25]^ The results of these studies were consistent with the findings of this study. Dietary education strategies need individualized advice to improve nutrient and diet quality, particularly those regarding fiber, fruit, and vegetable intake. Given the extensive benefits of adequate fruit and vegetable intake in those with chronic kidney disease on acidosis, weight management, and mortality, they play a critical role in food-based dietary prescriptions for people with chronic kidney disease.^[26]^

### 3.2. Analysis of the effect of dietary and nutritional nursing on the micro-inflammatory status of PD patients

The results showed that whole-process diet-based nutritional management for patients undergoing PD effectively improved their micro-inflammatory status (*P* < .05). A multicenter parallel trial showed that the oral administration of iron to malnourished PD patients can improve their malnutrition and micro-inflammatory statuses.^[27]^ However, there was low medication compliance among patients,^[28]^ making it difficult for patients to take their medications on schedule and in the recommended dose. Additionally, the out-of-pocket payment for oral iron administration increased the financial burden on patients. The micro-inflammatory status also affected the gastrointestinal function of patients and reduced their appetite and food intake, thus exacerbating the malnutrition and micro-inflammatory statuses of patients.^[29]^ Micro-inflammation in patients with PD is currently treated with chemical drugs, such as levocanidine, compound α-keto acid, and atorvastatin, and herbal medicines, such as ginseng and atractylodis macrocephalae compound granules.^[13,30–32]^ In fact, current treatments rarely involve dietary regimens. Exercises, such as Baduanjin, can also effectively reduce micro-inflammation in these patients. These results are consistent with the findings of this study.

### 3.3. Analysis of the importance of nurses in the management of patients with PD

The PD nurse visited the participants every month to assess whether their home environment was disinfected, hand hygiene was performed, and disinfection tools were qualified. To lower the risk of peritonitis, the nurse advised the participants on their home diet and provided calculations for daily caloric needs, caloric distribution, and other matters. Nutritional intake and status in patients undergoing peritoneal dialysis may be challenging to assess and correct. Evaluating patients frequently and regularly not only ensures patient education but also maintains adequate macronutrient and micronutrient intake to decrease morbidity and mortality and improve the quality of life.^[33]^ The results of a French cohort study showed that the assessment of PD patients by PD nurses is crucial for self-care outcomes, indicating that PD nurses play a critical role in the treatment of patients with PD.^[34]^ It is crucial for patients undergoing PD to continue care under the supervision of a PD nurse after discharge. However, there is no uniform standard for assessment and evaluation by PD nurses; therefore, there is a need for more studies to clarify the assessment criteria used by PD nurses.^[35]^

The “patient-centered” approach used in this study involved physicians, nurses, patients, and patients’ family members. The healthcare providers developed individualized diet plans to increase patients’ appetite and also offered dietary and exercise programs for each patient. These interventions decreased inflammation and the risk of peritonitis and improved the nutritional status of patients undergoing PD. They also emphasized the significance of self-care while reducing the financial burden on patients and boosting their compliance with self-care practices.

### 3.4. Relationship between the inflammatory state and nutrition in patients undergoing peritoneal dialysis

Our study found that the nutritional status of patients undergoing peritoneal dialysis is negatively correlated with the micro-inflammatory state. The better the patient’s nutritional status, the lighter the micro-inflammatory state. The results of this study were similar to those of Hu Shaolan et al.^[36]^ One possible reason for this phenomenon is that patients with malnutrition often exhibit elevated levels of inflammation. Malnutrition can impair vascular endothelial function, aggravate oxidative stress, and enhance the production of inflammatory factors. Conversely, various inflammatory factors are upregulated in the presence of inflammatory conditions, resulting in the loss of appetite, inadequate protein intake, and accelerated muscle protein breakdown, which finally contributes to malnutrition.^[37]^At the same time, the lack of the trace element zinc can also changes patients’ micro-inflammatory and affective states.^[38]^ Our research team’s personalized list for patients with peritoneal dialysis not only supplements the necessary protein intake but also enhances the consumption of trace elements. This approach enables patients to obtain various essential nutrients and trace elements from their diet, thereby improving their nutritional and inflammatory status.

Limitations of this study: This single-center study was conducted on patients admitted to a tertiary care hospital in Wuhan; therefore, the findings should be cautiously generalized to other populations. Additionally, since the total number of participants in this study was 111, the study sample cannot represent the whole population of patients with PD. As this paper does not refer to residual renal function, it might have partly affected the results of this study.

Prospect: If circumstances allow, the authors would like to devote more time and money to conducting parallel controlled trials on larger samples in this region or other regions.

## 4. Conclusion

Collaboration between the medical and nursing teams in the management of the entire diet of PD patients can improve malnutrition and micro-inflammation status and prevent peritonitis in PD.

## 5. Strengths and limitations

This single-center study was conducted on patients admitted to a tertiary care hospital in Wuhan; therefore, the findings should be cautiously generalized to other populations. Additionally, since the total number of participants in this study was 111, the study sample cannot represent the whole population of patients with PD.

Prospect: If circumstances allow, the authors would like to devote more time and money to conducting parallel controlled trials on larger samples in this region or other regions.

## Acknowledgments

The authors would like to thank the staff of the Dialysis Center and Department of Nephrology who helped us with data collection and others who provided their advice and support in this research project. The authors would like to express their gratitude to EditSprings (https://www.editsprings.cn) for the expert linguistic services provided.

## Author contributions

**Data curation:** Yani Lv, Li Guo.

**Investigation:** Yani Lv, Li Guo.

**Writing – original draft:** Peng Shu.

**Writing – review & editing:** Fang Xu.

## Supplementary Material



## References

[1] Lees JS Welsh CE Celis-Morales CA. Glomerular filtration rate by differing measures, albuminuria and prediction of cardiovascular disease, mortality and end-stage kidney disease. Nat Med. 2019;25:1753–60. 31700174 10.1038/s41591-019-0627-8 PMC6858876

[2] Zhao ZH Liu DW Pan SK. Epidemiological survey of peritoneal dialysis in Henan Province from 2013–2018. Chin J Kidney Dis. 2019;35:136–8.

[3] Qi HJ Zhang Y Jiang J. Progress in the care of malnutrition in peritoneal dialysis patients with end-stage renal disease. Gen Pract Nurs. 2022;20:1345–8.

[4] Piccoli GB Lippi F Fois A. Intradialytic nutrition and hemodialysis prescriptions: a personalized stepwise approach. Nutrients. 2020;12:785. 32188148 10.3390/nu12030785 PMC7146606

[5] Cui L Gong R. Effect of nutritional supplementation on mortality in peritoneal dialysis patients: a meta-analysis. Ther Apher Dial. 2023;27:296–303. 36071661 10.1111/1744-9987.13918

[6] Wu WQ Jin W. Effect of nutritional status on microinflammatory status and left heart function in peritoneal dialysis patients. Chin J Integr Chin West Med Nephrol. 2018;19:915–7.

[7] Zhang FP Li HP Wu FH. Panimmune-inflammation value is associated with poor prognosis in patients undergoing peritoneal dialysis. Ren Fail. 2023;45:2158103. 36632816 10.1080/0886022X.2022.2158103 PMC9848369

[8] Li J Lu WJ Gong SH. Research progress of microinflammatory state in peritoneal dialysis patients with chronic renal failure. J Nanchang Univ. 2021;61:80–3.

[9] Liu CL Jin YJ Han XL. Effects of intestinal microecological agents on residual renal function, microinflammation and oxidative stress in elderly peritoneal dialysis patients with chronic renal failure. Pract Geriatrics. 2021;4:358–61.

[10] Jin JW Zhang LM Zhang YJ. Effect of bailing capsule and prostil combined with peritoneal dialysis for chronic renal failure and the effects on residual renal function, inflammatory factors and nutritional status. J Liberation Army Med. 2020;32:81–5.

[11] Niu TM Han Y Luan XF. Effects of aerobic exercise combined with resistance exercise on microinflammation and T-cell subsets in peritoneal dialysis patients. Chin J Pract Internal Med. 2020;40:493–496,501.

[12] Tang ST Xu J Wang LY. Effects of atorvastatin combined with irbesartan on oxidative stress and microinflammatory status in elderly peritoneal dialysis patients. Mod Adv Biomed. 2019;19:3908–3911,3919.

[13] Hu SL Yang M Bai YH. Effect of compounded α-keto acid combined with low protein diet on nutrition and microinflammatory status of peritoneal dialysis patients. J Kunming Med Univ. 2019;40:106–10.

[14] Zhang XL Liu XS Fu LZ. Effects of remote dietary recording method and paper-based 3-D dietary recording method on adherence to nutritional management in patients with chronic kidney disease. Chin Family Med. 2021;24:1909–14.

[15] Ikizler TA Cuppari L. The 2020 updated KDOQI clinical practice guidelines for nutrition in chronic kidney disease. Blood Purif. 2021;50:667–71. 33652433 10.1159/000513698

[16] Pan XL Wang HL. Practical Diagnostic Medicine. Beijing: People’s Medical Publishing House; 2017.

[17] Chen J Peng H Yuan Z. Combination with anthropometric measurements and MQSGA to assess nutritional status in Chinese hemodialysis population. Int J Med Sci. 2013;10:974–80. 23801883 10.7150/ijms.5811 PMC3691795

[18] Nguyen B Bui QTH Tran PQ. Survival rates in elderly patients on continuous ambulatory peritoneal dialysis. int J Nephrol Renovasc Dis. 2023;16:131–41. 37155487 10.2147/IJNRD.S397555 PMC10122850

[19] Kolak E Radić J Vučković M Bučan Nenadić D Begović M Radić M. Nutritional and hydration status and adherence to dietary recommendations in Dalmatian dialysis patients. Nutrients. 2022;14:3553. 36079811 10.3390/nu14173553 PMC9460881

[20] Sahathevan S Se CH Ng S. Clinical efficacy and feasibility of whey protein isolates supplementation in malnourished peritoneal dialysis patients: a multicenter, parallel, open-label randomized controlled trial. Clin Nutr. ESPEN. 2018;25:68–77. 29779821 10.1016/j.clnesp.2018.04.002

[21] Luo Y Huang Y Zhang Y Xiang J Wu Q. Effect of nurse-led food exchange intervention for patients undergoing peritoneal dialysis . Clin Nephrol. 2020;93:140–8. 31939347 10.5414/CN109898

[22] Luan YH Peng WJ Zheng QY. Impact of a nurse specialist-led home follow-up program on peritoneal dialysis patients’ self-care ability. Nurs Pract Res. 2022;19:2592–7.

[23] Tao X Zhang H Lai L. A 12-week personalized physical activity and dietary protein intervention for older adults undergoing peritoneal dialysis: a feasibility study. Geriatr Nurs. 2022;47:247–53. 36007425 10.1016/j.gerinurse.2022.07.021

[24] Luo Y Yang Z Li H Chen X Huang Y. Effect Peng Liveness of a video-based exercise program on nutritional status and quality of life of peritoneal dialysis patients: a pilot randomized controlled trial. Clin Nephrol. 2023;99:105–17. 36519939 10.5414/CN110868

[25] Peng L Gao Y Lu R Zhou R. Efficacy of Omaha system-based nursing management on nutritional status in patients undergoing peritoneal dialysis: a randomized controlled trial protocol. Medicine (Baltim). 2020;99:e23572. 10.1097/MD.0000000000023572 PMC7748208 33371086

[26] Lambert K Ryan M Flanagan J. Dietary patterns, dietary adequacy and nutrient intake in adults commencing peritoneal dialysis: outcomes from a longitudinal cohort study. Nutrients. 2024;16:663. 38474791 10.3390/nu16050663 PMC10935117

[27] Hevilla F Padial M Blanca M. Effect on nutritional status and biomarkers of inflammation and oxidation of an oral nutritional supplement (with or without probiotics) in A multicenter randomized clinical trial “Renacare Trial”. Front Nutr. 2023;10:1107869. 36819685 10.3389/fnut.2023.1107869 PMC9936863

[28] Yao X Peng FN Dong WZ. Adherence to phosphorus binding agents and factors influencing phosphorus binding in elderly patients on continuous ambulatory peritoneal dialysis. Chin J Gerontol. 2022;42:1747–9.

[29] Wang H-B Yao Y-Z. Mechanism of PEW occurrence and pharmacological intervention in PD patients in microinflammatory state. Chin J Integr Chin West Med Nephrol. 2017;18:1126–8.

[30] Zhang WJ Zhao EY Zhang WZ. Efficacy of levocanidine combined with automated peritoneal dialysis in the treatment of diabetic nephropathy in elderly individuals. J Xinxiang Med Coll. 2022;39:865–70.

[31] Du K. Effect of atorvastatin combined with probucol on microinflammatory status, residual renal function and lipids in peritoneal dialysis patients. Hebei North College; 2019.

[32] Lv Y Zhang L Song B Wang YP. Clinical study on the treatment of persistent ambulatory peritoneal dialysis combined with protein–energy depletion in spleen deficiency and stagnation type with addition of ginseng lingbaijusan. J Nanjing Univ Tradit Chin Med. 2022;38:193–8.

[33] Kiebalo T Holotka J Habura I Pawlaczyk K. Nutritional status in peritoneal dialysis: nutritional guidelines, adequacy and the management of malnutrition. Nutrients. 2020;12:1715. 32521626 10.3390/nu12061715 PMC7352713

[34] Guillouët S Boyer A Lanot A Ficheux M Lobbedez T Béchade C. Assessment for assisted peritoneal dialysis by peritoneal dialysis nurses: results of a cohort study. Am J Nephrol. 2019;50:489–98. 31671419 10.1159/000503622

[35] Shi JJ Li X Gao ZY. A survey on the work and training status of peritoneal dialysis nurses in 157 hospitals. J Nurs. 2021;36:51–3.

[36] Hu SL Yang M Bai YH. Effects of malnutrition and microinflammatory status on cardiovascular calcification in peritoneal dialysis patients. J Kunming Med Univ. 2018;39:47–51.

[37] Graterol Torres F Molina M Soler-Majoral J. Evolving concepts on inflammatory biomarkers and malnutrition in chronic kidney disease. Nutrients. 2022;14:4297. 36296981 10.3390/nu14204297 PMC9611115

[38] Chan GC-K Ng JK-C Cheng PM-S Chow K-M Szeto C-C Li PK-T. Dietary micronutrient intake and its relationship with the malnutrition–inflammation–frailty complex in patients undergoing peritoneal dialysis. Nutrients. 2023;15:4934. 38068792 10.3390/nu15234934 PMC10707898

