# Supplementary material for: Effects of individualized dietary programs provided by nurses on nutrition and micro-inflammation of patients undergoing peritoneal dialysis (PD): A non-randomized controlled study
Source: Medicine (Baltimore). 2024 Nov 29;103(48):e40724. doi: 10.1097/MD.0000000000040724 (PMC11608727; doi:10.1097/MD.0000000000040724)

| peritoneal patient meal diary | | | | | |
| --- | --- | --- | --- | --- | --- |
| Named |  | Genders |  | Age(year) |  |
| Height(cm) |  | Daily protein(g) |  | Energy (Kcal) |  |
| Type of diet |  | | | | |
| Breakfast |  | | | | |
| Lunch |  | | | | |
| Dinner |  | | | | |
| Assessment nurses |  | Assessment of dietitians: |  | Date: |  |
| Note: |  | | | | |

1.Personalized dietary checklist for peritoneal dialysis patients.

2.An example of a dietary checklist for peritoneal dialysis patients:


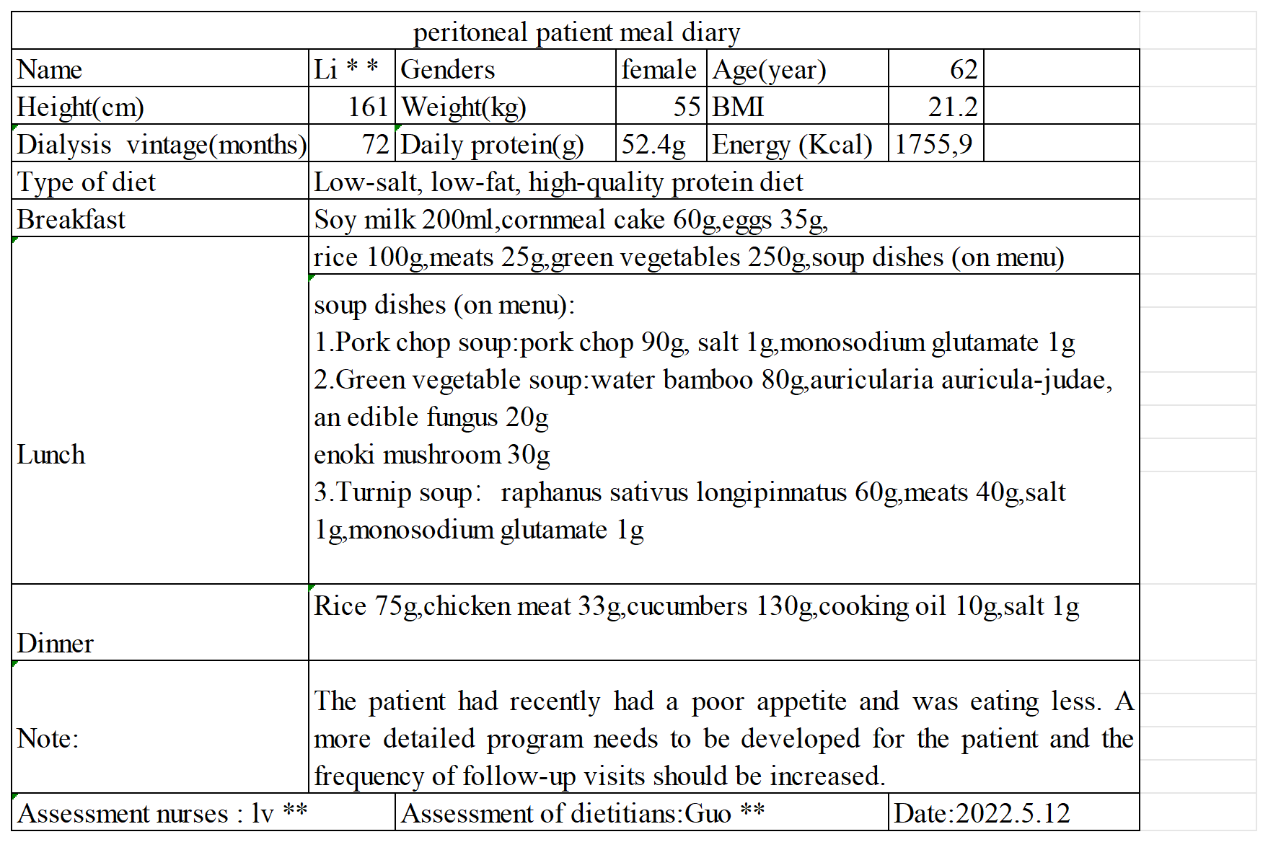

Supplement: Supplementary file 1 [file medi-103-e40724-s001.docx]
